# Revealing the Neural Mechanism Underlying the Effects of Acupuncture on Migraine: A Systematic Review

**DOI:** 10.3389/fnins.2021.674852

**Published:** 2021-05-20

**Authors:** Lu Liu, Tian Tian, Xiang Li, Yanan Wang, Tao Xu, Xixiu Ni, Xiao Li, Zhenxi He, Shan Gao, Mingsheng Sun, Fanrong Liang, Ling Zhao

**Affiliations:** ^1^Acupuncture and Tuina School, Chengdu University of Traditional Chinese Medicine, Chengdu, China; ^2^Acupuncture & Chronobiology Key Laboratory of Sichuan Province, Chengdu, China

**Keywords:** acupuncture, migraine without aura, functional neuroimaging, review, neural mechanism

## Abstract

**Background:** Migraine is a chronic neurological disorder characterized by attacks of moderate or severe headache and various neurological symptoms. Migraine is typically treated by pharmacological or non-pharmacological therapies to relieve pain or prevent migraine attacks. Pharmacological therapies show limited efficacy in relieving headache and are often accompanied by adverse effects, while the benefits of acupuncture, a non-pharmacological therapy, have been well-documented in both the treatment and prevention of acute migraine attacks. However, the underlying mechanism of the effect of acupuncture on relieving migraine remains unclear. Recent advances in neuroimaging technology have offered new opportunities to explore the underlying neural mechanism of acupuncture in treating migraine. To pave the way for future research, this review provides an overview neuroimaging studies on the use of acupuncture for migraine in the last 10 years.

**Methods:** Using search terms about acupuncture, neuroimaging and migraine, we searched PubMed, Embase, Cochrane Central Register of Controlled Trials, and China National Knowledge Infrastructure from January 2009 to June 2020 for neuroimaging studies that examined the effect of acupuncture in migraine. All published randomized and non-randomized controlled neuroimaging studies were included. We summarized the proposed neural mechanism underlying acupuncture analgesia in acute migraine, and the proposed neural mechanism underlying the sustained effect of acupuncture in migraine prophylaxis.

**Results:** A total of 619 articles were retrieved. After removing reviews, meta-analyses, animal studies and etc., 15 articles were eligible and included in this review. The methods used were positron emission computed tomography (PET-CT; *n* = 2 studies), magnetic resonance spectroscopy (*n* = 1), and functional magnetic resonance imaging (fMRI; *n* = 12). The analyses used included the regional homogeneity (ReHo) method (n = 3), amplitude of low frequency (ALFF) method (*n* = 2), independent component analysis (ICA; *n* = 3), seed-based analysis (SBA; *n* = 1), both ICA and SBA (*n* = 1), Pearson's correlation to calculate functional connectivity (FC) between brain regions (*n* = 1), and a machine learning method (*n* = 1). Five studies focused on the instant effect of acupuncture, and the research objects were those with acute migraine (*n* = 2) and migraine in the interictal phase (*n* = 3). Ten studies focused on the lasting effect of acupuncture, and all the studies selected migraine patients in the interictal phase. This review included five task-based studies and 10 resting-state studies. None of the studies conducted a correlation analysis between functional brain changes and instant clinical efficacy. For studies that performed a correlation analysis between functional brain changes and sustained clinical efficacy, the prophylactic effect of acupuncture on migraine might be through regulation of the visual network, default mode network (DMN), sensory motor network, frontoparietal network (FPN), limbic system, and/or descending pain modulatory system (DPMS).

**Conclusion:** The neural mechanism underlying the immediate effect of acupuncture analgesia remains unclear, and the neural mechanism of sustained acupuncture treatment for migraine might be related to the regulation of pain-related brain networks. The experimental design of neuroimaging studies that examined the effect of acupuncture in migraine also have some shortcomings, and it is necessary to standardize and optimize the experimental design. Multi-center neuroimaging studies are needed to provide a better insight into the neural mechanism underlying the effect of acupuncture on migraine. Multi-modality neuroimaging studies that integrate multiple data analysis methods are required for cross-validation of the neuroimaging results. In addition, applying machine learning methods in neuroimaging studies can help to predict acupuncture efficacy and screen for migraineurs for whom acupuncture treatment would be suitable.

## Introduction

Migraine is an episodic, recurrent dysfunction of brain excitability that is characterized by attacks of moderate or severe unilateral throbbing and pulsating headaches (Noseda and Burstein, 2013). The most characteristic symptoms associated with migraine include photophobia, phonophobia, cutaneous allodynia, and gastrointestinal symptoms (Dodick, 2018a). The latest Global Burden of Disease Study showed that 1.25 billion people had migraine in 2017; migraine is the fifth most common condition in the world, and the seventh most disabling (Dodick, 2018b). A better understanding of the mechanisms underlying the effect of treatment will help to improve treatments and reduce the negative impact of migraine.

Migraine is typically treated by various pharmacological or non-pharmacological therapies to relieve pain or prevent migraine attacks (Tfelt-Hansen, 2013). Although commonly used drugs, such as triptans, ergotamine preparations, and barbiturates, have achieved positive therapeutic effects in the treatment of migraine (Farri et al., 2012; Ou et al., 2020), side effects, such as weight gain, fatigue, sleep disturbance, gastrointestinal intolerance, and medication overuse headache, are common in patients with long-term use (Silberstein et al., 2012; Tfelt-Hansen, 2013). Hence, non-pharmacological therapies with less adverse effects that have a supplementary and preventive action are needed. Acupuncture is one non-pharmacological therapy that has relatively few side effects, and is widely used for managing migraine, especially for drug-refractory patients (Xu et al., 2018; Chen et al., 2020). Indeed, many studies have shown that acupuncture has acute analgesic and prophylactic effects on migraine without obvious side effects (Li et al., 2012; Zhao et al., 2017; Govind, 2019).

Numerous studies have suggested that migraine is largely a disorder of brain structure and function, and that the most notable symptom is persistent impairment of brain function (May, 2007; Webb et al., 2019). Migraine without aura (MWoA) is the most common subtype of migraine, accounting for about 80% (Steiner et al., 2013). Different subtypes of migraine have different pathogenesis and pathological changes (Kincses et al., 2019). Therefore, only including MWoA helps us to analyze the abnormal brain structure and function of these patients, and the regulation of abnormal brain function by acupuncture. Furthermore, advances in neuroimaging techniques have provided significant new insights into the neural mechanism underlying the effect of acupuncture on MWoA (Tedeschi et al., 2013). Lots of studies have helped to explore the regulation effect of acupuncture on abnormal brain function and functional network in MWoA (Lakhan et al., 2013; Liu et al., 2018; Colombo et al., 2019). However, few literatures have systematically reviewed these studies. The existing reviews haven't completely analyzed the characteristics of neuroimaging study design and summarized the corresponding neuroimaging results after instant acupuncture and sustained acupuncture, respectively (Teggi et al., 2016; Younis et al., 2017). Thus, this review was conducted to better understand the neural mechanism underlying the effect of acupuncture on migraine and its clinical implications. In this review, we summarize the findings of neuroimaging studies on the effect of acupuncture on migraine that were published in the last 10 years, describe how these findings have helped to explore the underlying neural mechanisms, discuss the limitations of these studies, and propose avenues for future neuroimaging work to study the effect of acupuncture on migraine.

## Methods

### Eligibility Criteria

(1) Types of studies. This review included all published randomized and non-randomized controlled clinical studies that examined the effect of acupuncture in migraine. Meta-analyses, reviews, and animal studies were not considered. The language of included studies was restricted to English and Chinese.
(2) Participants. Patients met the classification criteria of the International Headache Society for a diagnosis of MWoA.
(3) Interventions. Manual acupuncture (MA) and electroacupuncture (EA).
(4) Search methods for the identification of studies. PubMed, Embase, Cochrane Central Register of Controlled Trials, and China National Knowledge Infrastructure were searched from January 2009 to June 2020 for relevant clinical trials. References of the selected publications and bibliographies of reviews (found during the first screening of publications) were also inspected for relevant articles. The search terms and search strategy are shown in Table 1. PRISMA guidelines were followed.

**Table 1:** Search terms and search strategy.

**1. MRI**
**2. fMRI**
**3. PET-CT**
**4. Magnetic resonance imaging**
**5. Functional magnetic resonance imaging**
**6. Positron Emission Computed Tomography**
**7. ALFF**
**8. ReHo**
**9. ICA**
**10. FC**
**11. ROI**
**12. Amplitude of Low Frequency Fluctuations**
**13. Regional homogeneity**
**14. Independent component analysis**
**15. Functional connectivity**
**16. Region of interest**
**17. Blood oxygen level dependent**
**18. Neuroimaging**
**19. Brain**
**20. Acupuncture**
**21. Needle**
**22. Electroacupuncture**
**23. Acupoint**
**24. Migraine**
**25. “Neuroimaging-related” terms 1 OR 2 OR 3 OR 4 OR 5 OR 6 OR 7 OR 8 OR 9 OR 10 OR 11 OR 12 OR 13 OR 14 OR 15 OR 16 OR 17 OR 18 OR 19**
**26. “Acupuncture-related” terms 20 OR 21 OR 22 OR 23**
**27. FINAL SEARCH TERMS 24 AND 25 AND 26 AND**

*MRI, magnetic resonance imaging; fMRI, functional magnetic resonance imaging; PET-CT, positron emission computed tomography; ALFF, amplitude of low frequency fluctuations; ReHo, regional homogeneity; ICA, independent component analysis; FC, functional connectivity; ROI, region of interest*.

### Study Selection

The title and abstract of all the studies retrieved through the search strategy were first screened using EndNote, and duplicates were removed. Two reviewers also independently screened all titles and abstracts according to the inclusion criteria. Disagreements were resolved by discussion or a third reviewer. Then, the full text of these potentially eligible studies were again independently screened for eligibility by two reviewers, and disagreements were resolved by discussion or a third reviewer.

### Data Extraction

The following data were extracted from each eligible study: (1) publishing data (author, year); (2) the trial designs of included studies (subject cohorts, sample size, randomization, follow-up, clinical outcome measures, neuroimaging technologies, task-based/resting-state study design, ictal/interictal condition, image acquisition time, and analysis method); (3) acupuncture manipulation (manipulation style, session, duration, retention time, acupoints, description of “de qi” sensation (soreness, numbness, distention, and heaviness) and qualifications of acupuncturists); (4) participants (baseline demographic characteristics, diagnostic/inclusion/exclusion criteria, psychological assessment, avoidance of scanning during female participants' menstruation period); and (5) neuroimaging results.

### Data Analysis

First, bibliometric analysis was used to analyze study characteristics of included studies. In addition, we assessed the risk of bias in the included studies. Then, we narratively summarized the neuroimaging results in terms of the neural mechanism of instant acupuncture analgesia, and of the sustained effect of acupuncture.

## Results

### Study Characteristics of Neuroimaging Studies on the Use of Acupuncture to Treat Migraine

**Figure 1:**
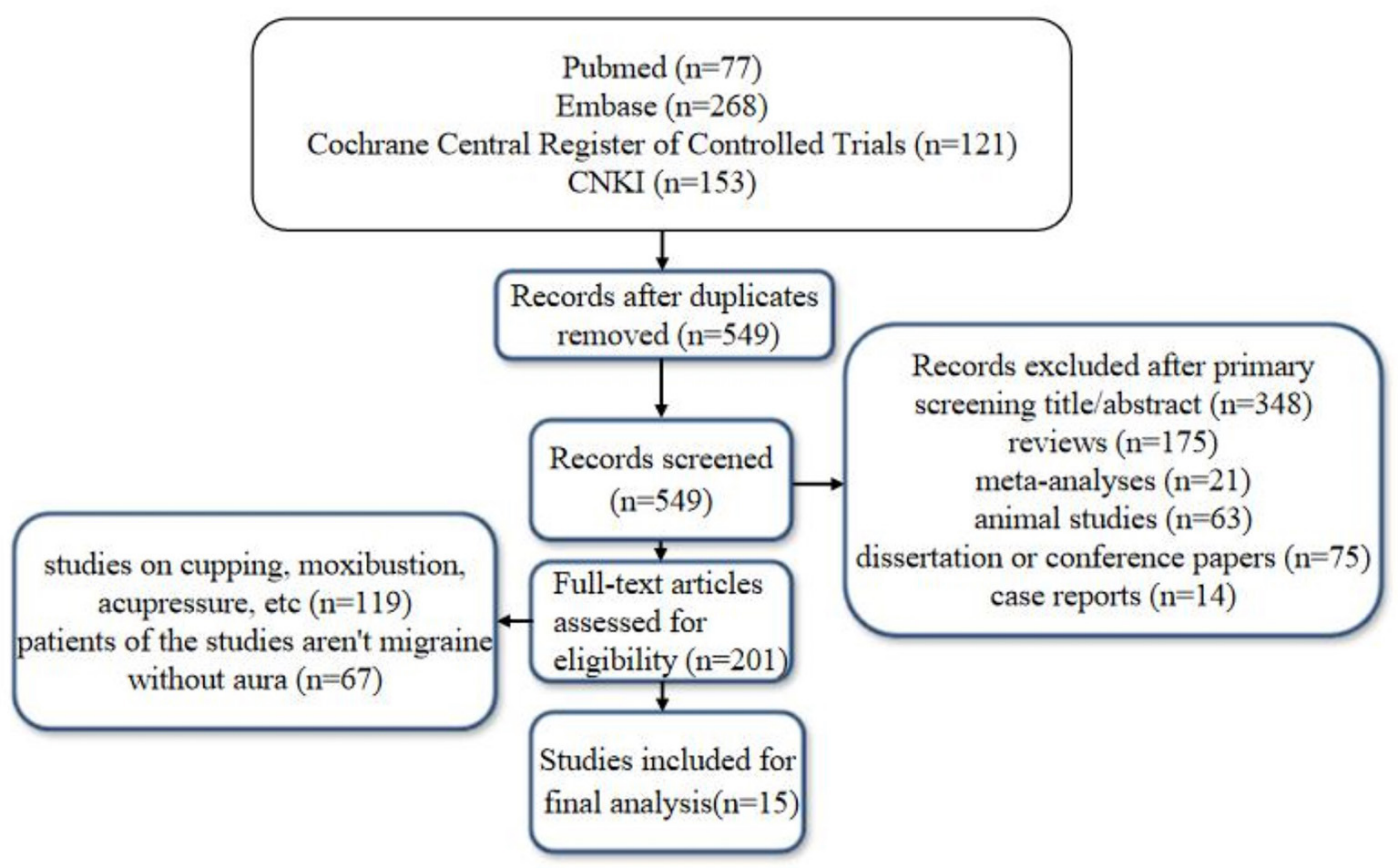
The flow diagram of literature screening.

**Figure 2:**
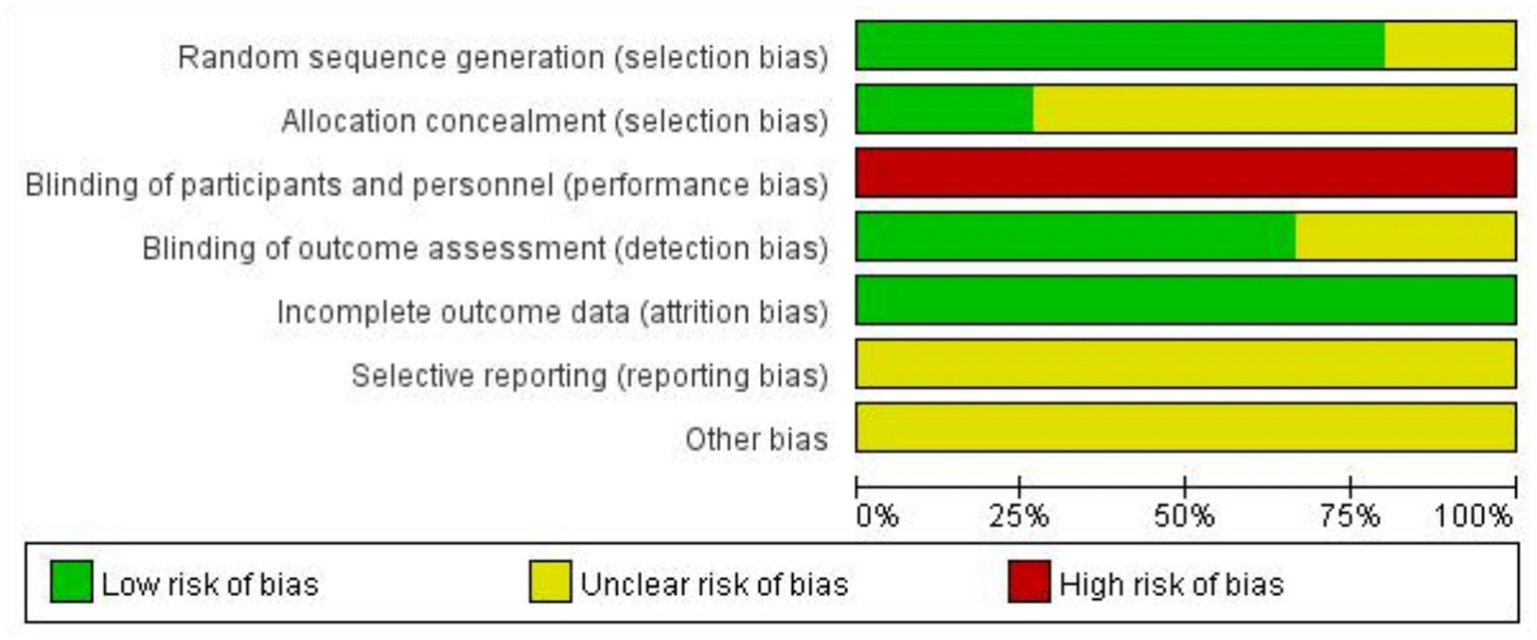
The assessment of risk of bias in the included literature.

The database search resulted in 619 studies; after removing duplicates, reviews, meta-analyses, animal studies, and trials for which no full text was available, a total of 15 papers were included for further evaluation. The flow diagram of literature screening could be found in Figure 1. The assessment of risk of bias in the included literature was shown in Figure 2.

#### Participants

A total of 634 patients with MWoA and 234 healthy volunteers were included in this review. Baseline demographic characteristics of the patients with MWoA and controls were comparable in the included studies. All studies stated their diagnostic, inclusion, and exclusion criteria clearly enough to enable us to only consider patients with MWoA. Of the 11 studies that included healthy controls (HCs), only 5 (45.5%) had strict inclusion/exclusion criteria for HCs. Only 4 (26.7%) studies assessed participants' anxiety and depression status using a self-rated anxiety scale and self-rated depression scale. Three (20%) studies avoided scanning during the menstrual period of female participants.

#### The Trial Designs and Analysis Method of Neuroimaging Studies

The sample sizes ranged from 10 to 72, and 8 (53.3%) studies had a sample size between 30 and 100. None of the included papers mentioned a sample size calculation. A total of 12 (80%) studies reported clinical outcomes. The clinical outcome measures were as follows: headache intensity (*n* = 11 studies), headache frequency (*n* = 9), duration of migraine attack (*n* = 3), self-rating anxiety and depression scales (*n* = 3), headache days (*n* = 2), amount of acute headache medications (*n* = 1), the Pittsburgh Sleep Quality Index (*n* = 1) and the HIT-6 questionnaire (*n* = 1). Eight (53.3%) studies carried out a correlation analysis between functional brain changes and clinical outcomes. There were two (13.3%) observational studies, five (33.3%) comparative studies, seven (46.7%) randomized controlled trials (RCTs), and 1 (6.7%) cross-sectional study. No follow ups focusing on functional brain changes of patients with MWoA after acupuncture treatment were made in included studies. PET-CT was used in 2 (13.3%) studies, MRS in 1 (6.7%) study, and fMRI in 12 (80%) studies; Of the fMRI studies, three (20%) employed regional homogeneity (ReHo) analysis, two (13.3%) used an amplitude of low frequency (ALFF) analysis, 3 (20%) used independent component analysis (ICA), 1 (6.7%) used seed-based analysis (SBA), 1 (6.7%) used both ICA and SBA, 1 (6.7%) used a machine learning method, and one (6.7%) used Pearson's correlation coefficient to calculate the functional connectivity (FC) between brain regions. Five studies focused on the instant effect of acupuncture; two of these selected patients with acute migraine attack as the research objects, and three selected patients with migraine in the interictal phase. Ten studies focused on the lasting effect of acupuncture, all of which selected patients with migraine in the interictal phase as the study subjects. Five (33.3%) studies used task-based (acupuncture stimulation) imaging, and 10 (66.7%) used resting-state imaging (before acupuncture treatment and after acupuncture treatment). Differences in the image acquisition time are shown in Supplementary Tables 2, 3.

#### Acupuncture Manipulation

Three (20%) studies used EA and 12 (80%) studies used MA. All included studies reported treatment frequency, duration of treatment, needle retention time in detail, but there were differences between the studies. Studies exploring instant acupuncture analgesia only treated one time. Studies exploring the sustained effect of acupuncture mostly treated five times a week, lasting for four weeks. A total of 21 acupoints were used. The most frequently used acupoints were SJ5 (Waiguan, *n* = 9 times), GB34 (Yanglingquan, *n* = 8), GB20 (Fengchi, *n* = 6), SJ8 (Sanyangluo, *n* = 5), GB33 (Xiyangguan, 5), GB41 (Zulinqi, *n* = 5) and GB40 (Qiuxu, *n* = 5). 14 (93.3%) studies recorded Deqi sensation, and eight (53.3%) studies introduced the qualifications of acupuncture operators.

### Potential Neural Mechanisms Underlying Instant Acupuncture Analgesia

#### Neuroimaging Results After Instant Acupuncture

**Figure 3:**
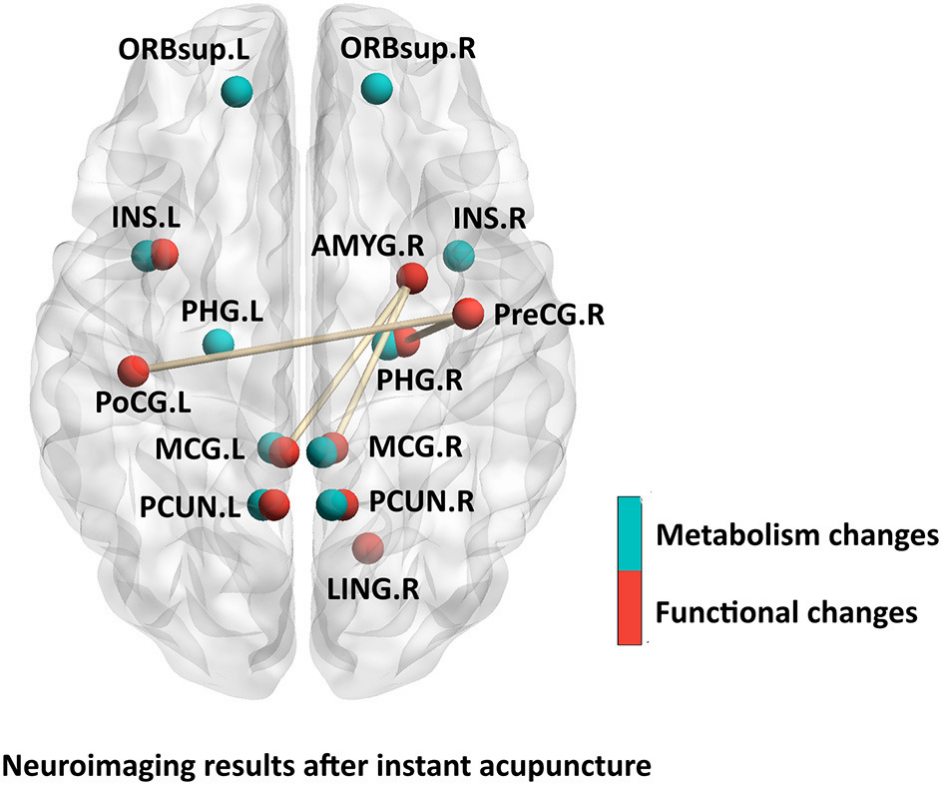
Neuroimaging results after instant acupuncture.

As shown in Supplementary Table 2 and Figure 3, two PET-CT studies selected patients with acute migraine attack as the research objects. After 30 min of acupuncture treatment, patients with MWoA showed increased metabolism in brain regions involved in the processing of pain including cognitive processing of pain [e.g., orbitofrontal cortex (Yang et al., 2012), the parahippocampal gyrus (Yang et al., 2014)] and affective-emotional processing of pain [e.g., the insula (Liu et al., 2016)]. Resting-state network, such as the DMN (e.g., the precuneus) and limbic system [e.g., the middle cingulate cortex (Yang et al., 2012, 2014)] showed increased metabolism after treatment. Three fMRI studies selected patients with MWoA in the interictal phase as the research objects. Using an FC analysis of fMRI data, Liu et al. reported decreased FC in pain-related brain networks (e.g., the right amygdala to the middle cingulate gyrus, the right precentral gyrus to the right parahippocampal gyrus, and the left postcentral gyrus to the right precentral gyrus); Decreased FC between these areas may underlie the pathogenesis of migraines, and it was increased by acupuncture at Zulinqi (GB41) (Liu et al., 2016). Ning et al. used an ALFF method to analyze fMRI data, and found decreased ALFF values in brain regions of the DMN (e.g., the inferior parietal lobule) and increased ALFF values in brain regions involved in the RFPN (right frontoparietal network; e.g., the right precentral gyrus and postcentral gyrus) in patients with MWoA after needling at GB41 (Ning et al., 2017). Another study revealed that ReHo value of the right lingual cortex differed between patients with MWoA and HCs during needling at GB41 (Han et al., 2017). The lingual gyrus in the occipital lobe has been identified as an important structure in the cortical spreading depolarization theory of migraine, this theory suggests that cortical spreading depression may cause the onset and persistence of migraine by activating trigeminal neurovascular system. The lingual gyrus is involved in the onset and persistence of migraine (Dahlem and Hadjikhani, 2009). Acupuncturing at GB41 may have a specific regulatory effect on the function of lingual gyrus in patients with MWoA, which may be one of the therapeutic mechanisms underlying the efficacy of acupuncture in treating migraine.

#### Correlation Between Neuroimaging Results and Clinical Outcomes

In 2012, we reported that the reduction of pain intensity was greater in the traditional acupuncture group (using specific acupoints of the Shaoyang meridians) than in the control group (using non-specific acupoints of Yangming meridians), and different patterns of cerebral glucose metabolism in the brain were observed in the two groups (Yang et al., 2012). In 2014, our team found that the reduction of pain intensity in the acupuncture group (using sub-specific acupoints on Shaoyang meridians) was slightly greater to that of the sham acupuncture group (using sham acupoint), but this difference was not significant. However, we found that the pattern of brain glucose metabolism change in the acupuncture group was targeted, while its distribution was disordered and random in the sham acupuncture group (Yang et al., 2014).

### Potential Neural Mechanisms Underlying the Sustained Effect of Acupuncture

It has long been acknowledged that the effect of acupuncture can be described in terms of the acute phase and the sustained phase. The curative function of acupuncture for migraine is not limited to instant cessation of acute pain (Li et al., 2009; Wang et al., 2012). Many studies have suggested that it is not only the immediate effect of acupuncture that is of interest, but also the delayed effect of the acupuncture-induced neural response (Bai et al., 2009; Liu et al., 2009, 2010; Zhang et al., 2009). We included 10 neuroimaging studies on the sustained effect of acupuncture in patients with MWoA in the interictal phase; the characteristics of these studies can be found in Supplementary Table 3. We investigated this sustained effect of acupuncture in migraine according to neuroimaging findings and the association of these with clinical outcomes.

#### Neuroimaging Results After Sustained Acupuncture

**Figure 4:**
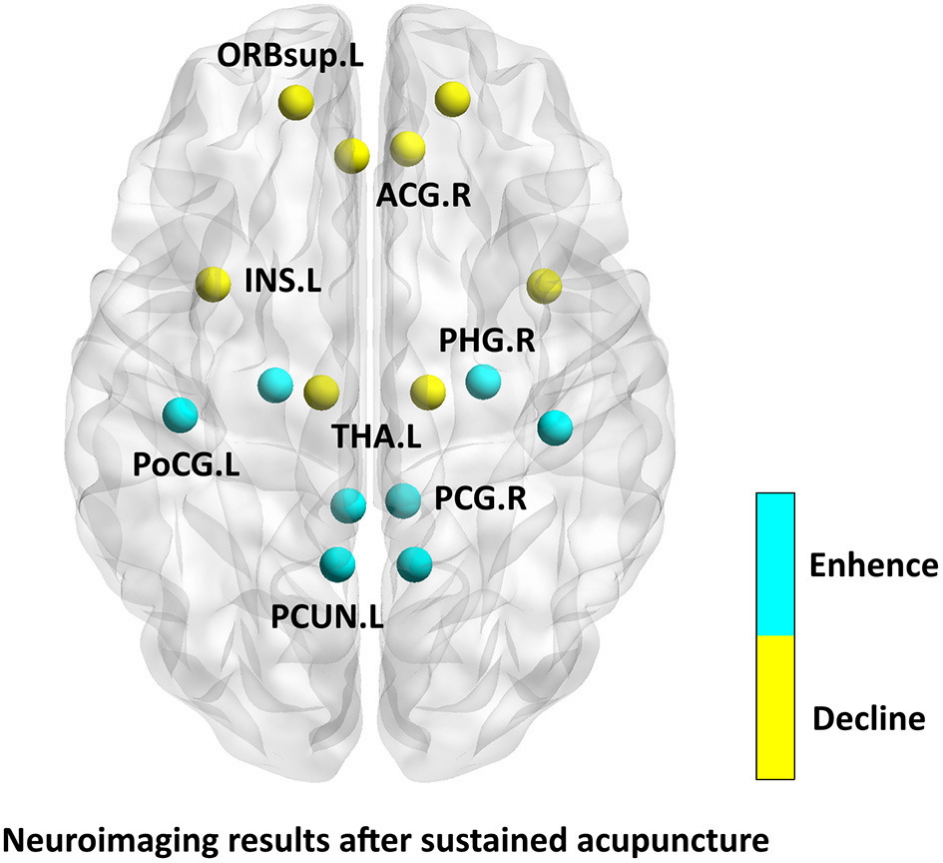
Neuroimaging results after sustained acupuncture.

As shown in Supplementary Table 3 and Figure 4, Researchers have observed increased ReHo/ALFF/brain metabolites after acupuncture treatment in brain regions involved in the processing of pain including the affective-emotional processing of pain [e.g., the insula (Zhao et al., 2014; Qin et al., 2019), cerebellum (Zhao et al., 2014), and brainstem (Zhao et al., 2014)], the cognitive processing of pain [e.g., the orbitofrontal cortex (Li et al., 2017a; Gu et al., 2018; Qin et al., 2019)] and the DPMS [e.g., the rostral ventromedial medulla/trigeminocervical complex (Li et al., 2017a)]. After treatment, researchers found decreased ReHo/ALFF/brain metabolites in brain regions involved in cognitive processing of pain [e.g., the hippocampus (Zhao et al., 2014; Qin et al., 2019)] and in resting-state network, such as, DMN [e.g., the posterior cingulate cortex (Zhao et al., 2014; Qin et al., 2019), precuneus (Zhao et al., 2014), and inferior parietal loblue (Zhao et al., 2014)], and FPN [e.g., the postcentral gyrus (Zhao et al., 2014; Qin et al., 2019)]. After long-term acupuncture treatment, an increased FC has been found within the DMN (Zhang et al., 2016; Zou et al., 2019) and FPN [e.g., the superior frontal gyrus and medial frontal gyrus (Zhang et al., 2016)]. An increased FC between the RFPN and the left FPN [e.g., the left precentral gyrus and left postcentral gyrus (Li et al., 2015)] and DMN [e.g., the left inferior parietal lobule (Li et al., 2015) and posterior cingulate cortex (Li et al., 2017b)] has been reported. Our team (Li et al., 2017b) observed decreased FC between the RFPN and DMN (the right precuneus) after acupuncture treatment. To explore the role of the right precuneus in acupuncture modulation, we performed an SBA using the right precuneus as a seed. We found increased FC between the DMN and DPMS (e.g., the rostral anterior cingulate cortex/medial prefrontal cortex), RFPN (e.g., dorsolateral prefrontal cortex and ventrolateral prefrontal cortex), and reward system (e.g., ventral striatum). In another study (Li et al., 2015), we chose the right ventrolateral periaqueductal gray as the seed, and found increased FC between the ventrolateral periaqueductal gray and limbic system (e.g., middle cingulate cortex), and the DPMS (e.g., rostral anterior cingulate cortex and left medial prefrontal cortex).

#### The Relationship Between Neuroimaging Results and Clinical Outcomes

Six studies found that ReHo/ALFF/brain metabolites/FC in certain brain regions were negatively correlated with headache intensity, as measured by visual Analog scale (VAS) scores after treatment (Zhao et al., 2014; Li et al., 2015, 2016, 2017a; Gu et al., 2018; Zou et al., 2019). The VAS scores was negatively correlated with average ReHo values in the anterior cingulate cortex and insula in the active acupoint group (Gu et al., 2018); another team also reported that brain metabolites in the bilateral thalamus was negatively correlated with decreased VAS scores (Gu et al., 2018). In another study, FC in the RFPN of patients with MWoA was negatively correlated with VAS scores after treatment (Li et al., 2015). In one study, we observed that changes in FC between ventrolateral periaqueductal gray and several brain regions, including the bilateral rostral anterior cingulate cortex, middle cingulate cortex, left superior frontal gyrus, thalamus, putamen, caudate, cerebellum, and middle frontal gyrus, were negatively associated with corresponding headache intensity changes after acupuncture treatment (Li et al., 2016). Another study from our team showed that ALFF values in the rostral ventromedial medulla/trigeminocervical complex were negatively correlated with VAS scores after treatment (Li et al., 2017a). Zou et al. found that after treatment FC within the DMN was negatively correlated with immediate VAS scores, and that increases in z-scores of the left precuneus were negatively correlated with reductions in the monthly amount of acute headache medications taken. Z-scores represents the standardized functional connectivity, and can be used for data processing and comparison (Zou et al., 2019).

One study found that FC was positively associated with VAS scores (Li et al., 2017b). In that study, we demonstrated that the decrease of RFPN FC between the bilateral precuneus, right paracentral gyrus, and postcentral gyrus was positively associated with a decrease in VAS scores. Recently, using FC analysis of fMRI data and a machine learning method, we found an association between headache frequency and changes in the neural marker which with abnormal FC within the visual network, DMN, sensorimotor network, and FPN in MWoA patients who received real acupuncture (Tu et al., 2020).

## Discussion

The beneficial effects of acupuncture for migraine have been widely recognized. Acupuncture not only reduces pain intensity of acute migraine attacks, but also prevents migraine by reducing the frequency of migraine attacks, acute relief medication intake, and pain intensity (May, 2007; Li et al., 2012; Webb et al., 2019). Changes in brain function detected by neuroimaging tools can be used as objective outcome measures to assess the clinical efficacy of acupuncture for migraine, and can give insights into the underlying mechanism. In the following, we will discuss the following three aspects: the status of designs given the existing studies and suggestions for future work, analysis of the current neuroimaging results and prospects for future studies.

### The Status of Designs Given the Existing Studies and Suggestions for Future Work

Our review revealed some limitations that could be addressed by future studies, as follows:

#### The Trial Designs and Analysis Method of Neuroimaging Studies

Only seven RCTs were included in this review. Although the number of neuroimaging studies of the use of acupuncture for migraine has been increasing, the relatively small number of studies may limit our conclusions. Most of the available neuroimaging studies investigating the effects of acupuncture on migraine were conducted on a relatively small scale. Small samples are common given the financial burden and additional constraints of neuroimaging studies, and this can lead to a low statistical power that might obscure significant results that could have clinical implications (Moayedi et al., 2018). To confirm the accuracy and repeatability of the current results in included studies and elucidate the complex neural mechanisms of acupuncture treatment, RCTs with larger sample sizes are needed. Statisticians have recently introduced several new, easily accessible fMRI-specific power analyses that make it easier to consider the desired effect and sample sizes (Mumford, 2012).

Only six studies reported that functional brain changes were associated with pain intensity, and two studies reported that functional brain changes were associated with headache frequency and medication use. It was unclear whether these studies performed correlation analyses between functional changes and other clinical outcomes; only positive results were reported. Future work should perform correlation analyses between functional brain changes and more clinical symptoms, such as pain intensity, frequency of attacks, use of medications, and symptoms of anxiety and depression, and fully report the results.

No neuroimaging scanning was performed during the follow-up period after acupuncture treatment in the current studies. Acupuncture has been reported to have a long-term therapeutic effect in the follow-up period after acupuncture treatment for migraine (Li et al., 2012). To assess the potential neural mechanism underlying the long-term efficacy of acupuncture on migraine prophylaxis, future work should not only consider the functional brain changes of patients with MWoA before and after acupuncture treatment, but also during the follow-up period.

Different neuroimaging techniques and analysis methods may make it hard to compare results across studies. Multi-modality imaging methods in larger patient populations may help to overcome some of these inconsistencies and improve our understanding of how different findings relate to each other (Schwedt et al., 2015; Russo et al., 2020).

Differences were found by fMRI in brain activity between the ictal and interictal visits in the brainstem/pons, thalamus, insula, cerebellum and cingulate cortex in migraine patients (Maleki et al., 2021). Another group noticed different activation patterns of the occipital cortex in migraine patients during ictal and interictal phase (Bramanti et al., 2005). Only two studies included in this review selected patients with acute migraine attacks as the research objects, and the rest 13 studies selected patients with migraine in the interictal phase as the study subjects. Migraine is a paroxysmal disease, and the onset of migraine is often unpredictable. Headache and neurological symptoms during migraine attack, time-consuming and noisy fMRI scanning have influenced patients' coordination and compliance to clinical research. These limitations make it difficult to conduct neuroimaging studies on spontaneous migraine attacks, and the number of related studies is relatively small. In the future, a large number of neuroimaging studies focusing on the effect of acupuncture for migraine during ictal phase are needed.

#### Participants

The diagnostic/inclusion/exclusion criteria for MWoA are clear. But the diagnostic/inclusion/exclusion criteria for HC vary greatly between studies. To improve the quality of research, more rigorous inclusion/exclusion criteria to define HCs are needed.

Participants' mental state has been reported to have a great impact on their brain function such as regional spontaneous neural activity and amplitude low-frequency oscillations (Liu et al., 2012, 2013). It has been reported that migraineurs with depression exhibit hypoechogenicity of the brainstem raphe, whereas those without depression exhibited hypoechogenic raphe (Tao et al., 2019). Of the studies included in this review, only four assessed participants' anxiety and depression status. Thus, future studies could employ a self-rated anxiety scale, self-rating depression scale, or the Beck Anxiety/Depression Inventory to assess participants' mental state and eliminate the confounding effect of depression/anxiety on neuroimaging results.

Changes of brain function and structure have been reported in women during menstruation (Hagemann et al., 2011; Veldhuijzen et al., 2013). However, only three studies ensured that female participants did not undergo neuroimaging during menstruation. In the future, researchers should optimize the neuroimaging detection scheme to ensure that female participants avoid receiving neuroimaging scanning if they are menstruating.

#### Acupuncture Manipulation

The differences in qualifications of acupuncturists, “de qi” sensation, manipulation methods, and frequency and duration of treatment affected the final results of the included studies. It could therefore be important to standardize acupuncture procedures, and define requirements for the angle, depth, and manipulation of acupuncture needles. In addition, ideally, a single professional acupuncturist should perform treatment within studies, and the “de qi” scale should be recorded.

Furthermore, to ensure that acupuncture treatment protocols used in neuroimaging studies are effective in migraine treatment, protocols that have been verified by clinical RCTs should be adopted.

### Analysis of the Current Neuroimaging Results After Sustained Acupuncture

The brain areas that had been changed after instant acupuncture mostly were the precuneus (Yang et al., 2012, 2014; Ning et al., 2017), the right precentral gyrus (Liu et al., 2016; Ning et al., 2017), the left postcentral gyrus (Liu et al., 2016), the right parahippocampal gyrus (Liu et al., 2016).

These brain regions are mainly located in the pain conduction pathway and somatosensory cortex, which are closely related to pain (Bornhövd et al., 2002; Akitsuki and Decety, 2009) and are the main pain regulating central system (Ploner et al., 1999). After summarizing the neuroimaging studies of acupuncture for migraine in the last 10 years, we found scientific evidence of acupuncture's immediate and sustained effect on migraine. None of the included studies performed a correlation analysis between functional brain changes and clinical outcomes immediately after acupuncture, although there seems to be a link between functional brain changes and clinical outcomes (Yang et al., 2012, 2014); nonetheless, the neural mechanism underlying the immediate effect of acupuncture analgesia remains unclear. The experimental design of studies that have examined immediate acupuncture analgesia also have some shortcomings. For example, the predisposing factors to migraine attacks and their symptoms and severity are not yet clear. Headache and neurological symptoms during migraine attack, time-consuming and noisy fMRI scanning have affected patients' coordination and compliance to clinical trials. These limitations make it difficult to use neuroimaging to assess the neural mechanism of immediate acupuncture analgesia.

### Analysis of the Current Neuroimaging Results After Instant Acupuncture

After sustained acupuncture treatment, the relief of pain intensity might be regulated by the DMN, FPN, limbic system and DPMS (Zhao et al., 2014; Li et al., 2015, 2016, 2017a,b; Gu et al., 2018; Zou et al., 2019). The reduction in the monthly amount of acute headache medications taken might be regulated by the DMN (Zou et al., 2019), and the reduction of headache frequency might be regulated by the DMN, visual network, sensory motor network, and FPN (Tu et al., 2020). The DMN is an important resting-state network, which consists of precuneus, posterior cingulate gyrus and etc (Raichle, 2006). The DMN has been associated with adaptive behavior, and cognitive, emotional, and attention processing (Raichle et al., 2001). Key components of the DMN are significantly related to individual pain sensitivity (Goffaux et al., 2014), are also involved in the “medial pain system,” which is considered to respond to pain stimuli (Zhuo, 2008)and plays an important role in pain processing (Kong et al., 2010; Loggia et al., 2013). The limbic system today is generally thought of as including the amygdala, the anterior cingulate cortex, the orbital and medial prefrontal cortex, the insula and etc (Price and Drevets, 2010). The limbic system is an important region for the processing of acute pain and anxiety (Maizels et al., 2012). The amygdala influences attention, conditioning, and memory retrieval, and plays a critical role in fear conditioning. The anterior cingulate cortex is primarily involved in modulating the affective component of pain, the anterior insula is involved when attention modulates pain. The prefrontal cortex serves a critical role in controlling pain modulatory circuits, specifically by driving endogenous pain-inhibitory circuits (Nagai et al., 2007). The frontoparietal region plays a dominant role in the formation and transmission of sensation, and connects the primary sensory area with the secondary sensory area (Lobanov et al., 2013). Other studies have demonstrated that the frontoparietal lobe is closely related to cognitive processing, memory working, and attention (Seeley et al., 2007). Accordingly, chronic continual pain, such as that experienced by patients with MWoA, invariably affects attention, and might alter FC of the FPN. The RFPN has been recognized as an important brain network for perception, somesthesis, and pain (Smith et al., 2009). Pain perception is modulated by the DPMS, allowing environmental, contextual and cognitive factors to influence our pain experiences (McMahon et al., 2013). The DPMS comprises a network of cortical and subcortical brain and brainstem regions that can inhibit nociceptive afferent brain inputs (Ossipov et al., 2010; Tracey, 2010; Goksan et al., 2018). Its integrated function is essential for effective modulation of sensory input to the central nervous system and behavioral responses to pain (Zhuo and Gebhart, 1997). From assessing neuroimaging evidence of the effect of acupuncture on pain intensity, analgesic medication use, and headache frequency, we can infer that the neural mechanism of sustained acupuncture treatment for migraine might be related to the regulation of pain-related brain networks. But the experimental design of studies that have examined the effect of sustained acupuncture also have some shortcomings. For example, there are differences in acupoint selection, frequency and duration of treatment. There's no unified standard for neuroimage acquisition time. Whether women should avoid menstrual period for MRI scanning, and whether the psychological state of migraine patients is evaluated. These factors affect the repeatability and scientificity of the studies, and bring difficulties to explore the neuroimaging mechanism of acupuncture's sustained effect.

### Prospects for Future Studies

In 3 studies, Liu et al. found that the interindividual variability of brain structure could predict the outcomes of an 8-week sham acupuncture treatment in patients with MWoA (Liu et al., 2017a,b, 2019). Recently, Yang et al. used a machine learning classification method to establish a predictive model of acupuncture efficacy in patients with MWoA based on pretreatment gray matter structure. The model had an 83% accuracy rate in distinguishing acupuncture responders from non-responders (Yang et al., 2020). These studies demonstrate that neuroimaging tools can be used in future work to not only evaluate clinical efficacy, but also to predict clinical efficacy. Indeed, the use of neuroimaging could provide an objective biomarker for acupuncture treatment responses of patients with MWoA and aid the development of personalized treatment. Also, the prediction of acupuncture responses could reduce medical costs for patients identified as probable non-responders, and using a diagnostic neural marker can be useful in patients who are unable to communicate or provide reliable self-reports. Further research is needed to verify whether these neuroimaging markers can be regarded as predictors of the effect of acupuncture on migraine, and whether other such predictive neuroimaging markers exist. To do this, future studies could use more recent techniques to comprehensively analyze neuroimaging data, such as machine learning methods. Machine learning is a sub-field of artificial intelligence, with the goal of learning a function from a set of data points through optimization of a certain performance metric, such as prediction accuracy. Machine learning methods are now key tools for neuroimaging data analysis in the field of pain research (Rashidi et al., 2019), and future work could consider using this method to better interpret neuroimaging findings on acupuncture treatment for migraine.

## Limitations

The present study has several limitations that should be noted. First, despite our thorough research, the number of neuroimaging studies into the effect of acupuncture on migraine was quite limited, which may limit our study findings. Second, although this review provides a detailed, structured overview of the current neuroimaging literature on the use of acupuncture to treat migraine, we identified data evaluation issues that can be difficult to avoid in neuroimaging and acupuncture research. Namely, every individual neuroimaging device, analysis method, and acupuncture intervention comes with the risk of bias such as performance bias and detection bias, which in turn could have biased our review.

## Conclusions

The neural mechanism underlying the immediate effect of acupuncture analgesia remains unclear, and the neural mechanism of sustained acupuncture treatment for migraine might be related to the regulation of pain-related brain networks. The experimental design of neuroimaging studies that examined the effect of acupuncture in migraine also have some shortcomings, and it is necessary to standardize and optimize the experimental design. Multi-center neuroimaging studies are needed to provide a better insight into the neural mechanism underlying the effect of acupuncture on migraine. Multi-modality neuroimaging studies that integrate multiple data analysis methods are required for cross-validation of the neuroimaging results. In addition, applying machine learning methods in neuroimaging studies can help to predict acupuncture efficacy and screen for migraineurs for whom acupuncture treatment would be suitable.

## Data Availability Statement

The original contributions presented in the study are included in the article/Supplementary Material, further inquiries can be directed to the Corresponding author.

## Author Contributions

This work was carried out as a collaboration between all authors. LL, TT, and XL wrote the first draft of the manuscript. LZ revised the first draft of the manuscript. YW, XL, XN, SG, and ZH collected the data. All authors have seen and agreed on the final submitted version of the manuscript.

## Conflict of Interest

The authors declare that the research was conducted in the absence of any commercial or financial relationships that could be construed as a potential conflict of interest.
