# Supplementary material for: Revealing the Neural Mechanism Underlying the Effects of Acupuncture on Migraine: A Systematic Review
Source: Front Neurosci. 2021 May 20;15:674852. doi: 10.3389/fnins.2021.674852 (PMC8172773; doi:10.3389/fnins.2021.674852)
Supplement: Supplementary file 1 [file Table_1.DOCX]

Migraine is a chronic neurological disorder characterized by attacks of moderate or severe headache and various of neurological symptoms. Acupuncture is well-documented in both the treatment and prevention of acute migraine attacks. However, the underlying mechanism of the effect of acupuncture on relieving migraine remains unclear. Advances in neuroimaging techniques have provided significant new insights into the neural mechanism underlying the effect of acupuncture on migraine. These neuroimaging studies have helped to explore the regulation effect of acupuncture on abnormal brain function and functional network in migraine. However, few literatures have systematically reviewed these studies. The existing reviews haven’t completely analyzed the characteristics of neuroimaging study design and summarized the corresponding neuroimaging results after instant acupuncture and sustained acupuncture respectively. Thus, this review was conducted to better understand the neural mechanism underlying the effect of acupuncture on migraine and its clinical implications. In this review, we summarize the findings of neuroimaging studies on the effect of acupuncture on migraine that were published in the last 10 years, describe how these findings have helped to explore the underlying neural mechanisms, discuss the limitations of these studies, and propose avenues for future neuroimaging work to study the effect of acupuncture on migraine.
