# Supplementary material for: Revealing the Neural Mechanism Underlying the Effects of Acupuncture on Migraine: A Systematic Review
Source: Front Neurosci. 2021 May 20;15:674852. doi: 10.3389/fnins.2021.674852 (PMC8172773; doi:10.3389/fnins.2021.674852)
Supplement: Supplementary file 2 [file Table_2.docx]

Supplementary Table 2 Neuroimaging results after instant acupuncture

| **Publishing data** | | **Sample size** | **Neuroimaging technologies** | **Image acquisition time** | **Neuroimaging results** |
| --- | --- | --- | --- | --- | --- |
| **Author** | **Year** |  |  |  |  |
| Yang  et al[23] | 2012 | 30  (MWoA) | 18FDG-PET-CT  (task-based: acupuncture stimulation) | <24 hours from the previous migraine attack to the beginning of the scan; acquired image data  sets at 40 minutes after the 18F-FDG injection. | TAG vs. MG: *increased metabolism* in cortical regions (MTC, OFC, insula, MFG, angular gyrus, PCC, precuneus, MCC). *Decreased metabolism* in cortical regions (parahippocampus, hippocampus, fusiform gyrus, postcentral gyrus) and cerebellum.  CAG vs. MG: *increased metabolism* in cortical regions (MTC, supratemporal gyrus, supramarginal gyrus, and MCC). *Decreased metabolism* in cerebellum. |
| Yang  et al[24] | 2014 | 30  (MWoA) | 18FDG-PET-CT  (task-based: acupuncture stimulation) | <24 hours from the previous migraine attack to the beginning of the scan; acquired image data  sets at 40 minutes after the 18F-FDG injection. | AG vs. MG: *increased metabolism* in cortical regions (MFG, postcentral gyrus, the precuneus, parahippocampus, MCC.) and cerebellum. *Decreased metabolism* in cortical regions (MTC).  SAG vs. MG: *increased metabolism* in cortical regions (PCC, insula, inferior temporal gyrus, MTC, superior temporal gyrus, postcentral gyrus, fusiform gyrus, inferior parietal lobe, superior parietal lobe, supramarginal gyrus, middle occipital lobe, angular and precuneus). *Decreased metabolism* in cortical regions (parahippocampus) and cerebellum. |
| Liu  et al[25] | 2016 | 28(15MWoA,13HC) | Task-based fMRI  (FC) | >24 hours from the previous migraine attack to the beginning of the scan;  resting state: 8min 10s; during needling:  10min 10s; structural  image scanning: 5 min. | AG before needling vs. HC before needling:*decreased FC* in left parahippocampal gyrus to right posterior cingulate gyrus.  AG after needling vs. AG before needling: *increased FC* in the same side precentral gyrus to postcentral gyrus, left postcentral gyrus to right precentral gyrus, right precentral gyrus to right parahippocampal gyrus, right amygdala to middle cingulate gyrus, left hippocampus to left supramarginal gyrus and right hippocampus.  AG after needling vs. HC after needling: *decreased FC* in left parahippocampal gyrus to posterior cingulate gyrus and angular gyrus. |
| Han  et al[27] | 2017 | 20  (10MWoA,10HC) | Task-based fMRI  (ReHo) | acquired image data in 9 min 10s after needling. | AG before needling vs. HC before needling:*increased ReHo value* in cortical regions (bilateral superior frontal gyrus, MFG, medial superior frontal gyrus, straight gyrus, left medial superior frontal gyrus and right inferior frontal gyrus). *Decreased ReHo value* in cortical regions (right middle occipital gyrus, superior occipital gyrus, cuneiform lobe, anterior cuneiform lobe and cortex around rectangular fissure).  HC after needling vs. HC before needling: *decreased ReHo value* in cortical regions (left MFG, superior frontal gyrus, precentral gyrus)  AG after needling vs. AG before needling: *decreased ReHo value* in cortical regions (left MFG, superior frontal gyrus, inferior frontal triangle).  AG after needling vs. HC after needling: right lingual as the *specific ReHo value* region between AG and HC after needling. |
| Ning  et al[26] | 2017 | 32(16MWoA,16HC) | Task-based fMRI  (ALFF) | during needling: 1min 10s; resting state: 9  min. | AG after needling vs. AG before needling: *increased ALFF values* in the cortical regions (right precentral and postcentral gyri) and *decreased ALFF values* in the cortical regions (bilateral precuneus, right MFG and right inferior parietal lobule).  AG after needling vs. HC after needling: *increased ALFF values* in the cortical regions (right precentral, postcentral gyri, hippocampus, MTG, STG) and *decreased ALFF values* in the cortical regions (left precuneus, calcarine, cuneus, parietal gyrus). |

MWoA, migraine without aura; HC, healthy controls; TAG, traditional acupuncture group; CAG, control acupuncture group; SAG, sham acupuncture group; MG, migraine group; AG, acupuncture group; fMRI, functional magnetic resonance imaging; PET-CT, positron emission tomography combined with computed tomography; ALFF, amplitude of low frequency fluctuations; ReHo, regional homogeneity; FC, functional connectivity ; MTC, middle temporal cortex;OFC, orbital frontal cortex; MFG, middle frontal gyrus; PCC, posterior cingulate cortex; MCC, middle cingulate cortex; STG, superior temporal gyrus; MTG, middle temporal gyrus;
