# Supplementary material for: Revealing the Neural Mechanism Underlying the Effects of Acupuncture on Migraine: A Systematic Review
Source: Front Neurosci. 2021 May 20;15:674852. doi: 10.3389/fnins.2021.674852 (PMC8172773; doi:10.3389/fnins.2021.674852)
Supplement: Supplementary file 3 [file Table_3.docx]

Supplementary Table 3 Neuroimaging results after sustained acupuncture

| **Publishing data** | | **Sample size** | **Neuroimaging technologies** | **Image acquisition time** | **Neuroimaging results** |
| --- | --- | --- | --- | --- | --- |
| **Author** | **Year** |  |  |  |  |
| Zhao  et al[34] | 2014 | 40(MWoA) | rsfMRI  (ReHo) | patients didn’t suffer from a migraine  attack at least 72 hours prior to the brain scan; fMRI scans were performed on each group at the baseline and after 8 weeks’ treatment. | AG after treatment vs. AG before treatment: *increased ReHo values* in cortical regions (bilateral ACC, insula, STG, SMA, cuneus, lingual gyrus), subcortical structures (thalamus), cerebellum, brainstem. *Decreased ReHo values* in cortical regions (PCC, MFG, angular gyrus, precuneus, MTG, left hippocampus, inferior parietal lobule, inferior temporal gyrus, right postcentral gyrus).  Inactive acupoint group after treatment vs. inactive acupoint group before treatment: i*ncreased ReHo values* in cortical regions (left ACC, medial frontal gyrus).*Decreased ReHo values* in the cortical regions (right MFG).  AG vs. inactive acupoint group: *increased ReHo values* in cortical regions (ACC, STG, SMA). *Decreased ReHo values* in the cortical regions (hippocampus, MFG, and MTG), and subcortical structures (thalamus). |
| Zhang  et al[39] | 2015 | 24(12MWoA,12HC) | rsfMRI  (ICA) | patients didn’t suffer from a migraine  attack at least 72 hours prior to the brain scan; the acupuncture treatment should  begin within 3 days after the first fMRI scanning and  the second scanning should be done within 3 days after the acupuncture treatment course ended. | AG before treatment vs. HC: *decreased FC* in the bilateral superior frontal gyrus, medial frontal gyrus, inferior parietal lobule, ACC, cingulate gyrus, PCC, supramarginal gyrus, precuneus, MFG, inferior frontal gyrus, STG, MTG.  AG after treatment vs. AG before treatment: *increased FC* in the bilateral superior frontal gyrus, medial frontal gyrus, precuneus, inferior parietal lobule, posterior cingulate cortex, cingulate gyrus, STG, MTG, supramarginal gyrus.  AG after treatment vs. HC: *decreasd FC* in the bilateral superior frontal gyrus, MFG, medial frontal gyrus, inferior parietal lobule, ACC, cingulate gyrus, precuneus, and supramarginal gyrus. |
| Li et al[41] | 2015 | 24(12MWoA,12HC) | 1.rsfMRI(ICA)  2.DTI | patients didn’t suffer from a migraine  attack at least 72 hours prior to the brain scan; the acupuncture treatment should  begin within 3 days after the first fMRI scanning and the second scanning should be done within 3 days after the acupuncture treatment course ended. | AG before treatment vs. HC: *decreased FC* with the RFPN in the left precentral gyrus, the left supramarginal gyrus, the left inferior parietal lobule, and the left postcentral gyrus  AG after treatment vs. AG before treatment: *increased FC* with the RFPN in the left precentral gyrus, the left inferior parietal lobule, and the left postcentral gyrus. |
| Li  et al[43] | 2015 | 104(62MWoA,42HC) | rsfMRI  (SBA) | patients didn’t suffer from a migraine  attack at least 72 hours prior to the brain scan; MRI scans were applied at the end of the forth and eighth weekends for migraine patients. | MG vs. HC: *increased rs-fc* between the ventrolateral PAG and the bilateral adjacent PAG. *Decreased rs-fc* between the ventrolateral PAG and the bilateral mPFC, OFC, rACC.  AG+SAG after treatment vs. AG+SAG before treatment: *increased rs-fc* between ventrolateral PAG and the bilateral MCC, rACC and left mPFC.  AG vs.SAG: *increased rs-fc* between ventrolateral PAG and the bilateral ventral/anterior mPFC, left middle occipital gyrus/cunues, right middle occipital gyrus/cunues. |
| Li  et al[42] | 2016 | 118(72MWoA,46HC) | rsfMRI  (ICA) | patients didn’t suffer from a migraine  attack at least 72 hours prior to the brain scan; MRI scans were applied at the end of the forth and eighth weekends for migraine patients. | MG vs. HC: *decreased rs-fc* between the RFPN with the bilateral precuneus, lingual gyrus, MTG, STG, left fusiform, secondary somatosensory cortex (S2), right cerebellum, inferior occipital gyrus, inferior temporal gyrus and cuneus; *decreased rs-fc* between right precuneus with the left precuneus, supramarginal gyrus, and inferior temporal gyrus.  AG+SAG after treatment vs. AG+SAG before treatment: *increased rs-fc* between the RFPN with the bilateral PCC. *Decreased rs-fc* between the RFPN with the right precuneus and left MFG; *increased rs-fc* between right precuneus the bilateral rACC/mPFC, ventral striatum, middle/inferior occipital gyrus, cuneus, dorsolateral PFC and cerebellum, and left ventrolateral PFC, right STG. |
| Li  et al[38] | 2017 | 104(62MWoA,42HC) | rsfMRI(ALFF) | patients didn’t suffer from a migraine  attack at least 72 hours prior to the brain scan; MRI scans were applied at the end of the forth and eighth weekends for migraine patients. | MG vs. HC: *increased ALFF value* in cortical regions (left posterior insula and left putamen/caudate), *decreased* *ALFF value* in cortical regions (bilateral middle occipital cortex/cuneus and RVM/TCC).  AG after treatment vs. AG before treatment: *increased ALFF value* in cortical regions (bilateral OFC, bilateral RVM/TCC), bilateral rostral midbrain. D*ecreased ALFF value* in cortical regions (left middle occipital cortex/cuneus).  AG vs. MG: *increased ALFF value* in cortical regions (the bilateral OFC and bilateral RVM/TCC).  AG vs. SAG: *increased ALFF value* in the cortical regions (bilateral RVM/TCC). |
| Gu  et al[37] | 2018 | 45(15MWoA,15HC,15CH) | MRS | before and after acupuncture treatment | AG before acupuncture vs. AG after acupuncture:  *N-acetylaspartate/creatine increased* in subcortical structures (bilateral thalamus)  HC before acupuncture vs. HC after acupuncture: no difference  CH before acupuncture vs. CH after acupuncture: no difference |
| Zou  et al[40] | 2019 | 32(14MWoA,18HC) | rsfMRI  (ICA,SBA) | within 2 days before and after acupuncture treatment. | AG before treatment vs. HC: *reductions in* the left superior prefrontal gyrus, left precuneus and *decreased* DMN z-scores within the two regions. *Decreased FC* between the right temporal lobe and left ACC, between the right temporal lobe and bilateral precuneus, between the the right temporal lobe and bilateral superior medial gyrus, between the right temporal lobe and bilateral superior prefrontal gyrus, between the right temporal lobe and left temporal lobe.  AG before treatment vs. AG after treatment: *increase FC* between the right temporal lobe and left ACC, between the right temporal lobe and bilateral superior medial gyrus, between the right temporal lobe and bilateral precuneus. |
| Qin  et al[36] | 2019 | 40(MWoA) | rsfMRI  (ReHo) | before and after acupuncture treatment; not during a migraine attack | AG before treatment vs. AG after treatment: *increased ReHo value* in the cortical regions(anterior cingulate cortex, precentral gyrus, OFC, insula, inferior parietal lobule, pontine nucleus, cerebellar tonsils and orbital frontal inferior gyrus), subcortical structures (ventral lateral nucleus and ventral posteromedial nucleus of the thalamus, posterolateral nucleus of thalamus). D*ecreased ReHo values* in the cortical regions (postcentral gyrus, posterior cingulate cortex, left precentral gyrus, hippocampus), right brain bridge.  SAG before treatment vs. SAG after treatment: *increased ReHo value* in the cortical regions (right tongue gyrus, the left anterior lobe, the anterior cingulate cortex, the lower occipital gyrus), *decreased ReHo value* in the subcortical structures (left ventral posterolateral nucleus of the thalamus). |
| Tu  et al[44] | 2020 | 70(MWoA) | rsfMRI  (machine learning method) | patients didn’t suffer from a migraine  attack at least 72 hours prior to the brain scan; MRI scans were applied at the end of the forth and eighth weekends for migraine patients. | MG vs. HC: *the differences were located* primarily within the occipital lobe, including both occipital and postoccipital areas (middle occipital gyrus and calcarine); the sensorimotor network, including the parietal and postparietal(inferior parietal lobule) cortices; part of the medial-cerebellum; the cingulo-opercular network, including the anterior-insula, dorsal anterior cingulate cortex, medial frontal cortex, and thalamus; DMN, including the angular gyrus, fusiform gyrus, occipital gyrus; the frontal parietal network, including the anterior frontal cortex and ventral lateral prefrontal cortex.  AG vs. SAG vs. MG: we linked the changes in the connectome-based response to changes in headache frequency and found a significant correlation in AG but not in SAG or MG. |

AG, acupuncture group; SAG, sham acupuncture group; CH, chronic headache; fMRI, functional magnetic resonance imaging; rsfMRI, resting-state functional magnetic resonance imaging; ALFF, Amplitude of Low Frequency Fluctuations; ReHo, Regional homogeneity; FC, functional connectivity; ICA, independent component analysis; MRS, magnetic resonance spectrum; DTI, diffusion tensor imaging; OFC, orbital frontal cortex; MFG, middle frontal gyrus; PCC, posterior cingulate cortex; STG, superior temporal gyrus; MTG, middle temporal gyrus; rs-fc, resting-state functional connectivity; ACC, anterior cingulate cortex; SMA, supplementary motor area; DMN, default mode network; RFPN, right frontoparietal network; PAG, periaqueductal gray; rACC, rostral anterior cingulate cortex; mPFC, medial prefrontal cortex; PFC, prefrontal cortex; RVM / TCC, rostral ventromedial medulla / trigeminocervical complex; SBA, seed based analysis; MCC, middle cingulate cortex;
